# Patterns of motor signs in spinocerebellar ataxia type 3 at the start of follow-up in a reference unit

**DOI:** 10.1186/s40673-016-0042-6

**Published:** 2016-02-23

**Authors:** Irene Pulido-Valdeolivas, David Gómez-Andrés, Irene Sanz-Gallego, Estrella Rausell, Javier Arpa

**Affiliations:** Department of Anatomy, Histology and Neuroscience, School of Medicine, Universidad Autónoma de Madrid, C/ Arzobispo Morcillo 4, 28029 Madrid, Spain; Trastornos del Desarrollo y Maduración Neurológica (TRADESMA), IdiPaz-UAM, Madrid, Spain; Department of Neurology, Hospital Universitario La Paz, Madrid, Spain; Department of Pediatrics, Hospital Universitario Infanta Sofía, San Sebastián de los Reyes, Madrid, Spain; Department of Neurology, Complejo Asistencial de Ávila, Ávila, Spain

**Keywords:** Ataxia, Machado-Joseph disease, Multivariate analysis, Network analysis, SPECT, SARA scale

## Abstract

**Background:**

Spinocerebellar ataxia type 3 (SCA3) is a neurodegenerative disorder that affects the cerebellar system and other subcortical regions of the brain. As for other cerebellar diseases, the severity of this type of ataxia can be assessed with the Scale for Assessment and Rating of Ataxia (SARA) which gives a total score that reflects functional impairment out of 8 cerebellar function tests. SCA3 patients score profile is heterogeneous on at the start of follow up. This study investigates possible patterns in those profiles and analyses the impact of other usually concurrent signs of impairment of extracerebellar motor systems in that profile variability by means of multivariate statistical approaches.

**Methods:**

Seventeen patients with SCA3 underwent systematic anamnesis, neurological and SARA assessment, visual evaluation of ^123^I-Ioflupane (DaTSCAN) single-photon emission computed tomography (SPECT) imaging and electrophysiological studies (nerve conduction and electromyography). Patterns in the profiles of SARA item scores were investigated by hierarchical clustering after multivariate correspondence analysis. A network analysis was used to represent relationships between SARA item scores, clinical, genetic and neurological examination parameters as well as abnormalities of DaTSCAN SPECT imaging and electrophysiological studies.

**Results:**

The most frequently altered SARA items in all patients are gait and stance, and three profiles of SCA3 patients can be distinguished depending mainly on their degree of impairment in those two items. Other SARA items like the score on heel-shin slide contribute less to the classification. Network analysis shows that SARA item scores configure a single domain that is independent of the size of the mutated expanded allele and age of onset, which are, in turn closely and inversely correlated. The severity of cerebellar dysfunction is correlated with longer disease duration, altered visual evaluation of DaTSCAN SPECT imaging and decreased patellar reflexes. Neither the presence of pyramidal or extrapyramidal signs nor the intensity of polyneuropathy is correlated with the SARA items scores.

**Conclusions:**

Pattern recognition approaches are useful tools to describe clinical phenotypes of ataxias and to identify particular configurations of cerebellar signs.

**Electronic supplementary material:**

The online version of this article (doi:10.1186/s40673-016-0042-6) contains supplementary material, which is available to authorized users.

## Background

Spinocerebellar ataxia type 3 (SCA3 or Machado-Joseph disease) is an autosomal dominant neurodegenerative disorder, clinically expressed as a spectrum of progressive cerebellar dysfunctions variably associated with parkinsonism, pyramidal signs, neuropathy and external ophtalmoplegia, associated with atrophy of olivo-ponto cerebellar pathways and cerebellar cortex and frequently combined with lesions in the basal ganglia [1] and with abnormalities in the presynaptic dopaminergic pathway [2, 3]. SCA3 relies upon the expansion of CAG repeats in the ATXN3 gene which encodes for the cysteine protease ataxin-3 [1, 4]. The documented variability of the clinical phenotype [5] seems to rely in part on the number of CAG gene repeats which is strongly and negatively correlated with the age of disease onset [6]. The age of disease onset is very variable (from childhood to elderly) [7] and influences the frequency of clinical signs associated with cerebellar ataxia, such as abnormal tendon reflexes or decreased vibration sense [8, 9], but is not conditioning factor for the presence of other frequent concomitant signs such as supranuclear opthalmoplegia, myopathy, amyotrophy and sphincter and swallowing difficulties [9]. Another part of the phenotypical variability is related to the patient’s geographical origin [10].

Up to five clinical phenotypes have been classically distinguished on the basis of the age of onset, co-existent peripheral neuropathy and signs of alteration in extrapyramidal and pyramidal pathways [11], but not on the basis of different modular cerebellar dysfunction. The variability in cerebellar phenotypes of SCA3 and other spinocerebellar ataxias has not been systematically studied, although cerebellar function assessments of these patients do not seem to respond to an homogeneous profile [12]. This variability may reveal topographical heterogeneity of the neural lesions within the modular organization of the human cerebellum [13], and might follow patterns that can only be investigated by means of modern multivariate techniques. The definition of such clinical patterns in SCA3 and other spinocerebellar ataxias could contribute to a better comprehension of these diseases and to optimize the diagnostic process and care plan, especially at the start of follow-up when the patients first come to a reference unit.

Unravelling patterns of cerebellar dysfunction requires a systematic, valid and standardized clinical examination able to measure the severity of ataxia. The Scale for Assessment and Rating of Ataxia (SARA) is a reliable and widely used tool [14, 15] that assesses gait alteration, disequilibrium at stance, sitting instability, speech disturbance, limb dysmetria and coordination (dysdiadochokinesia), providing a total score which is calculated as the sum of all item scores and used to evaluate the severity of ataxia. The global score ranges from 0 to 40 points, which is the sum of single scores in gait (0–8 points), stance (0–6 points), sitting (0–4 points), speech disturbance (0–6 points), finger chase (0–4 points), nose-finger (0–4 points), fast alternating hand movements (0–4 points) and heel-shin slide (0–4 points). The higher the total value of the SARA score, the more severe the ataxia.

As previously mentioned above, SCA3 variably includes a diversity of abnormalities in other motor pathways that might influence the pattern of global motor dysfunction. The relationship of these abnormalities (measured either by clinical examination or by complementary tests) on the severity of ataxia has not been systematically defined.

This study attempts to classify a sample of SCA3 patients on the basis of possible patterns in the combination of single SARA item scores and the relationships of concomitant impairment of extracerebellar motor pathways by means of multivariate statistical approaches. In order to accomplish that, we have designed a descriptive study to 1) systematically describe the profiles and possible patterns of ataxia severity referred to individual SARA items scores in a sample of patients with genetically confirmed SCA3 at the beginning of the follow up in our reference unit, and 2) to study the relationships between single SARA items scores and visual evaluation of ^123^I-Ioflupane (DaTSCAN) single-photon emission computed tomography (SPECT) imaging, nerve conductions, electromyography and other clinical parameters.

## Results

### Descriptive analysis

The clinical assessments and single SARA scores of our 17 patients are reported in Tables 1 and 2. The median of the disease duration was 8 years (range: 0–21years) and the median age at onset was 38 years (range: 12–61years). Median of total SARA score was 11 points. The most severely affected patient had 27.5 points while the least affected one had 4 points. The number of repeats in expanded allele ranged from 50 to 70 (median 68). Decreased patellar reflex (DPR) was present in 53 % of patients. Pyramidal signs were found in 82.4 % of patients. 17.6 % of patients presented extrapyramidal signs and 76.5 % of patients had abnormalities in the visual evaluation of DaTSCAN SPECT imaging indicating an alteration in presynaptic dopamine pathway. 58.5 % of patients had polyneuropathy (5.9 % mild, 29.4 % moderate and 23.5 % severe).

**Table 1:** Description of clinical variables in a group of SCA3 patients

Patient	Age (years)	Sex (M/F)	Age at onset (years)	Evolution (years)	Repeats	Family with several patients in the study	Total SARA	Sensibility disturbance at clinical examination	Patellar arreflexia or hyporreflexia	Distal axonal polyneuropathy defined by electrophysiological studies	Pyramidal signs	Clinical extrapyramidal affection	Presynaptic abnormalities at DAT-SCAN
P1	18	M	12	6	NA	No	4	Distal hypopalesthesia	No	No	Yes	No	No
P2	71	F	61	10	65	A	16	Distal hypopalesthesia	Yes	Severe	Yes	No	Yes
P3	60	F	31	29	70	B	27.5	Tactile and thermoalgesic distal hypoesthesia	Yes	Moderate	Yes	Yes	Yes
P4	45	M	37	8	68	No	15.5	None	No	Mild	Yes	No	Yes
P5	73	M	45	28	66	No	13.5	Distal hypopalesthesia	Yes	Moderate	No	No	Yes
P6	60	M	60	0	65	A	8.5	Distal hypopalesthesia	Yes	Moderate	No	No	Yes
P7	34	F	25	9	NA	C	27	Distal hypopalesthesia	Doubtful	Moderate	Yes	No	Yes
P8	35	M	27	8	NA	B	7	None	No	No	Yes	No	Yes
P9	45	M	43	2	68	C	9	Global distal hypoesthesia	Yes	Severe	Yes	Yes	Yes
P10	68	F	56	12	66	No	14	Distal hypopalesthesia	Yes	No	No	No	Yes
P11	40	M	38	2	70	B	4	Distal hypopalesthesia	No	No	Yes	No	Doubtful
P12	28	F	23	5	75	No	11	Global distal hypoesthesia	No	No	Yes	No	Yes
P13	50	M	49	1	65	D	7	Global distal hypoesthesia	Yes	Moderate	Yes	Yes	NA
P14	48	M	40	8	69	D	13	None	Yes	Severe	Yes	No	Yes
P15	55	M	49	6	50	No	8.5	None	No	No	Yes	No	No
P16	40	M	30	10	73	No	11.5	None	No	No	Yes	No	Yes
P17	46	M	36	10	72	C	8.5	Global distal hypoesthesia	No	Severe	Yes	No	Yes

Polyneuropathy was assessed by clinical sensitivity examination, decreased patellar reflex and electrophysiological studies. Pyramidal signs were assessed by presence of plantar cutaneous reflex, Hoffman’s sign or clonus at triceps surae muscle. Extrapyramidal affection was defined either clinically (bradykinesia, rigidity, rest tremor or hypomimia) or by pre-synaptic abnormality in 123I-DaTSCAN SPECT imaging. “NA”: not acquired (in the case of number of repeats, the technique that was applied could be used for diagnosis but was not reliable to quantify the number of CAG repeats). An intermediate value was given to the doubtful interpretation in the DaTSCAN SPECT imaging. Patients from different families were included. If a subject is member of family with other subjects in this study, the membership is indicated by a code (family A, family B, family C and family D)

**Table 2:** Median and range of variables in our SCA3 sample of patients

	Abbreviation	Median	Range
Age (years)	---	46	18–73
Time of evolution (years)	EVO	8	0–29
Age at onset (years)	ONS	38	12–61
Numbers of repeats in expanded allele	REP	68	50–75
Total SARA score	---	11	4–27.5
Gait	GAI	3	1–8
Stance	STA	2	0–6
Sitting	SIT	1	0–4
Speech disturbance	SPE	1	1–4
Right finger chase	RFC	1	0–2
Left finger chase	LFC	1	0–2
Right nose-finger test	RNF	1	0–2
Left nose-finger test	LNF	1	0–2
Right fast alternating hand movements	RAM	1	0–3
Left fast alternating hand movements	LAM	1	0–3
Right heel-shin slide	RHS	1	0–2
Left heel-shin slide	LHS	1	0–2

### Pattern analysis

**Fig. 1:**
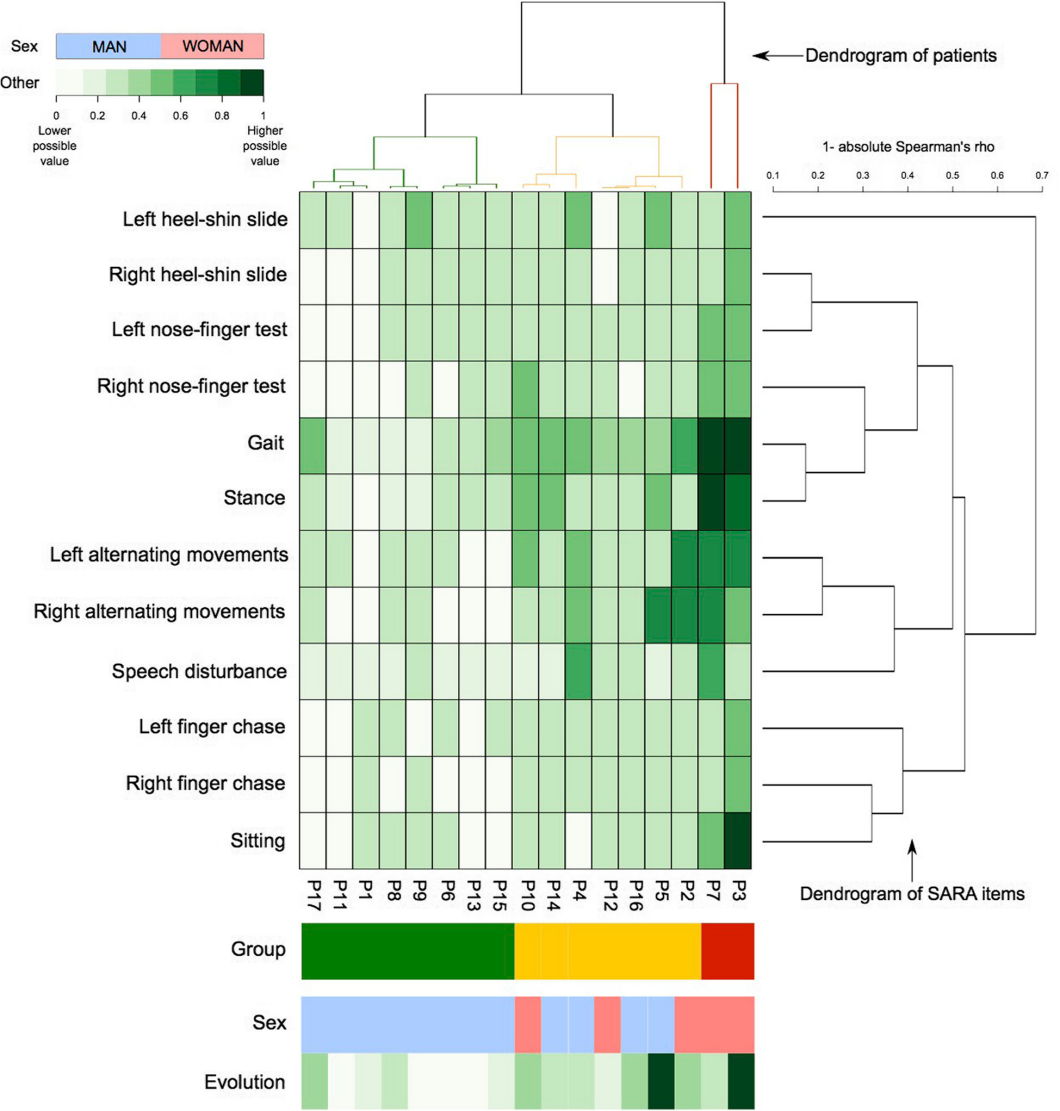
Heatmap that represents the variability of cerebellar dysfunction and its characteristic pattern for SCA3. Central heatmap represents the affection of each patient (ordered according the dendrogram of patients, *top*) in each SARA item (ordered according the dendrogram of SARA items, *right*). The closer the junction line between two elements is to heatmap in a dendrogram, the more similar they are. Each colour-coded cell represents the value for each SARA item in a particular patient, as shown in the top left scale. The lower cells represent the group of affection, sex and disease duration (years). Note three types of patients according to severity of cerebellar signs. Heatmap shows a fingerprint of cerebellar signs in SCA3: gait and stance are impaired in the majority of patients and, in contrast, other variables, are relatively more preserved in a significant proportion of patients

Figure 1 shows a heatmap that represents the variability of severity of ataxia and its characteristic pattern for SCA3 patients. The heatmap shows single values of SARA items for every patient. SARA items and patients are reordered following hierarchical clustering classification represented by dendrograms (classification of patients on the top of the heatmap and classification of SARA items on the right).

The dendrogram on the right side shows that SARA items are highly correlated between them as indicated by the general low values of “1 – absolute Spearman’s rho coefficient”.

Three clusters or groups of patients can be observed in the classification of patients as shown in the top horizontal superior dendrogram. The formation of these groups mainly depends on the severity of ataxia (reflected by the intensity of colour in the heatmap cells). The first group (green cluster in top dendrogram) is formed by those patients (P17, P11, P1, P8, P9, P6, P13 and P15 as indicated at the bottom line) with general milder impairment. The second group (orange cluster) is formed by those patients (P10, P14, P4, P12, P16, P5 and P2) that had moderate impairment. The third group (red cluster in top dendrogram) is formed by patients (P7 and P3) who showed severe ataxia.

Scores obtained in each SARA item influenced the classification but it can be observed that severity of ataxia increases with impairment of some individual SARA items (such as gait and stance tests) while other (such as heel-shin slide or nose-finger tests) are only moderately impaired even in the most severe cases of ataxia.

The formation of groups is very well related with the score in gait and stance tests. The more severe impairment in gait and stance, the higher probability of being classified in a more severely affected group of ataxia. In our sample, gait and stance are nearly always altered, even in patients with mild ataxia in the first group. Moreover, the impairment in alternating movement tests also has an effect in the classification of the severity of ataxia. These tests can remain normal or mildly affected in the first group, mildly or moderately affected in the second group and moderately affected in the two most severe cases. However the impairment in nose-finger, heel-shin slide, finger chases and sitting tests has a lower effect on classification. Patients in the first group show normal or mild affection in these tests while patients in the second group are generally mildly impaired. Patients with the most severe form of ataxia show moderate or severe abnormalities in those items. Although most of our patients suffered speech disturbance, it is usually mild in the whole sample with only occasional moderate affection in some cases. These results configure a sort of profile or pattern of the cerebellar dysfunction in SCA3 patients.

Disease duration and especially, sex also influence on our patients’ classification. Women seem to have a more aggressive form of the disease and patients with shorter disease duration classified into the first group.

**Fig. 2:**
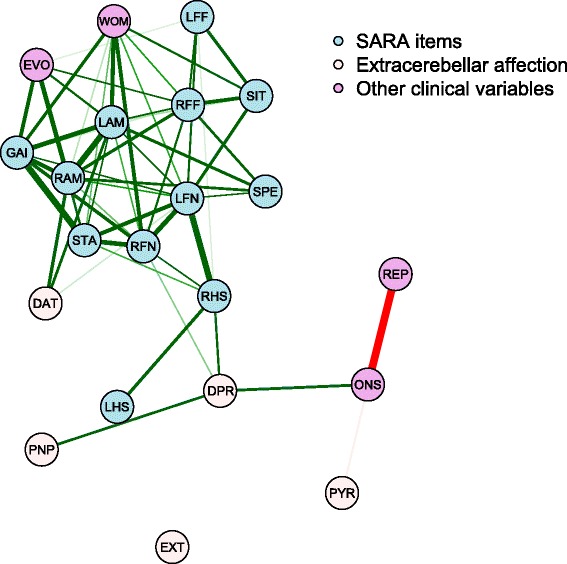
Graphical representation of network analysis. Blue circles represent SARA items, light purple circles are the clinical variables and variables taken from neurological examination are in light pink. Lines represent correlations between two parameters (green for positive and red for negative correlation). The wider the line joining two variables, the more closely correlated these two variables. For this analysis, gender has been transformed into a numerical variable (1 = woman, 0 = man) and has been accordingly represented by WOM. Number of repeats in the expanded allele (REP) and age at onset (ONS) are highly correlated in negative way. Women show a more altered gait, left alternating movement and right finger-to-nose. Disease duration (EVO) is positively associated with right and left alternating movements (RAM and LAM), gait (GAI), stance (STA –not clearly visible in the plot–) and right finger-to-finger (RFF). SARA items (blue) are densely interconnected with each other forming a clear merging main domain. The presence of visual abnormalities in DaTSCAN SPECT imaging (DAT) has a positive relation with right and left alternating movements (RAM and LAM). The presence of decreased patellar reflexes (DPR) has a positive correlation with right finger-nose test (RFN) and right heel-shin slide (RHS), and is positively correlated with the age at onset of disease (ONS) and the degree of polyneuropathy (PNP). The other clinical variables (pyramidal signs, clinical extrapyramidal affection and the presence of polyneuropathy) are independent of cerebellar dysfunction expressed by SARA items

Network analysis representation shows the relationship between the clinical variables and single SARA items scores (Fig. 2). SARA itemized scores are highly inter-correlated. This means that cerebellar dysfunction explored by different SARA items is organized in a single module, integrated in the same community without clear divisions. This suggests that the cerebellar motor abnormalities measured by SARA items in SCA3 nearly behave as a single element in which neurodegeneration seems to impair the whole spectrum of cerebellar motor function. In particular, the most closely correlated pairs of variables are stance with gait tests (rho = 0.828, *p* < 0.001).

The number of allelic CAG repeats is strongly and negatively correlated with age at onset (rho = − 0.914, *p* < 0.001), which indicates that patients with the more expanded allele are the ones with earlier start of the disease. It is remarkable that these two variables are barely correlated with single SARA item scores. However, disease duration is positively correlated with some SARA items, the longer duration, the more severe impairment, in particular for gait (rho = 0.684, *p* = 0.002), stance (rho = 0.596, *p* = 0.01), alternating movements (rho = 0.728, *p* < 0.001 for right side; rho = 0.583, *p* = 0.01 for left side) and finger chase (rho = 0.556, *p* = 0.02 for right side; rho = 0.507, *p* = 0.03 for left side) tests. These two last correlations are slightly asymmetric. Regarding sex of patients, women tend to have more difficulties performing left alternating movements (rho = 0.704, *p* = 0.002), right nose-finger test (rho = 0.687, *p* = 0.002), gait (rho = 0.645, p = 0.005) and sitting (rho = 0.585, *p* = 0.01) tests, this correlation being also slightly asymmetric. Alterations in presynaptic dopamine pathway as measured with visual inspection of DaTSCAN SPECT imaging are correlated with impairment in right and left alternating movements (rho = 0.658, *p* = 0.006 and rho = 0.62, *p* = 0.01 respectively) and left nose-finger test (rho = 0.51, *p* = 0.04). Patients with decreased patellar reflexes tend to have later onset of the disease (rho = 0.631, *p* = 0.007) and have more problems performing right finger-nose test (rho = 0.524, *p* = 0.02) and right heel-shin slide (rho = 0.6, *p* = 0.005). Asymmetries in the relationships of SARA items with decreased patellar reflex exist. We did not find any correlation between clinical extrapyramidal signs, pyramidal signs or severity of polyneuropathy with any of the single SARA items scores.

## Discussion

This study inquires into the variable manifestations of cerebellar dysfunction expressed by the items of SARA in patients with SCA3, and explores the influence of the coexisting of non-cerebellar motor symptoms and other personal and clinical variables in that variability.

We have used multivariate approaches to assess the clinical heterogeneity of SCA3 patients. These approaches are consolidated techniques in other disciplines [16], but they have not been widely used in studies devoted to ataxias for the definition of complex phenotypes. Network analysis representation summarizes relationships between parameters at a glance. Its interpretation is simple and intuitive. Mechanisms or modules can be easily detected. In contrast, classification of patients using multiple correspondence analysis is also important to better define the spectrum of dysfunction in a particular cerebellar disease. With this technique, classification is data-driven and objective, and representation of its results in heatmaps facilitates understanding of different clusters of concomitant signs as well as the relative contribution of each cerebellar sign to classification and enables data sharing in future meta-analytic approaches [17]. Multivariate approaches seem to be essential to improve pattern recognition in clinical settings and their underlying principles are included in daily diagnostic methods used intuitively by clinicians [18].

Our network analysis shows that cerebellar dysfunction in patients with SCA3 measured by the scores of SARA items is organized in a single integrated module. This means that the variety of motor cerebellar dysfunctions are closely interrelated, so that the variation in a cerebellar test should be accompanied with variations in the whole set of SARA items. Moreover, the single module of ataxia severity is independent of the number of CAG repeats of the expanded allele and of the age at disease onset. In contrast, it is dependent on disease duration, sex (being woman), decreased patellar reflexes and alteration of presynaptic dopaminergic pathway as referred to DaTSCAN SPECT imaging. These parameters have different impact in SARA cerebellar tests. For instance, disease duration influences some tests, being particularly related with gait, stance, finger chase and hand alternating movements, indicating that these parameters might be particularly useful to monitor disease progression. In addition, our results show that patients with greater allele expansion have earlier onset of disease and that cerebellar dysfunctions occur in frequency and intensity regardless of the concomitance of the impairment of pyramidal pathways, clinical involvement of the extrapyramidal system and severity of polyneuropathy.

Although clinical signs of affection of the extrapyramidal system have been frequently reported in patients with SCA3 [19], our results show that those are not correlated with the presence of visual abnormalities in DaTSCAN SPECT imaging. We hypothesize that subclinical damage of the presynaptic pathway occurs in a large proportion of SCA3 patients and that its traditional clinical expression (bradykinesia, hypomimia, rigidity and rest tremor) only shows up with major damage appears. Interestingly, our results show that even in the absence of clinical extrapyramidal signs, SCA3 patients may show qualitative abnormalities in visual evaluation of DaTSCAN SPECT imaging, especially those that have difficulties in tests implying fast alternating movements. The impairment of fast alternating movements is also an early and even preclinical sign of parkinsonism [20–22], suggesting that this sign is affected by subtle and common impairment of the presynaptic dopaminergic pathway in SCA3, while other traditional clinical extrapyramidal signs in SCA3 could require a more intense impairment of presynaptic pathway or other modifiers of disease expression that we have not explored in this study. Visual observation of DaTSCAN SPECT imaging is usually enough to detect the presence of abnormalities in this test. However, quantitative techniques may be an interesting tool in future research. Visual interpretation only differentiates normal from abnormal based on striatal shape, extent, symmetry and, specifically in SCA3 patients, intensity. Quantitative techniques provide with numerical parameters of all these features but they also have problems with reproducibility and interpretation [23]. Although one limitation of our study is the qualitative definition of DaTSCAN SPECT imaging by visual observation, we think that quantitative assessment of DaTSCAN SPECT imaging should be taken into account as a tool in future research.

The finding that the decrease of patellar reflexes (DPR) is related with a later onset of SCA3 disease has been previously reported [24]. DPR reflects an extreme form of nerve dysfunction and it is related with the intensity of polyneuropathy. The correlation between DPR and impairment in the right finger-nose and the right heel-shin slide tests could be explained by the importance of common subjacent proprioceptive processes in both phenomena. However, this fact merits further study.

The finding that the number of CAG allelic repeats is negatively correlated with the age at onset of disease is also consistent with previous reports [8, 24, 25]. Dürr et al. [8] described relationships between number of repeats and abnormal reflexes, loss of vibration sensitivity and polyneuropathy. Infante et al. [26] found higher frequency of pyramidal signs (hiperreflexia, spasticity and Babinski’s sign) in patients with longer gene expansions and Schmitz-Hübsch et al. [24] found longer repeats in patients with spasticity and hyperreflexia. However, in our sample, these variables or similar ones are not associated with longer gene expansions. These discrepancies could be explained by different statistical approaches, heterogeneity between samples and by differences in the definition of upper motor neuron lesion (in our study pyramidal signs were assessed by presence of extensor plantar cutaneous reflex, Hoffman’s sign or clonus at triceps surae muscle).

The relationship between sex and cerebellar dysfunction in SCA3 has not received much attention apart from the fact that inherited paternal mutant allele is more unstable [1]. In our sample of patients, women are more severely affected than men, but we cannot rule out that our effect is only a consequence of biased distribution between women and men and of other factors that influence on the severity of cerebellar dysfunction. For instance, women in our study tend to have longer disease duration. However, we cannot exclude sex-related mechanisms in the ataxia phenotype.

We have objectively defined three groups of SCA3 patients mainly on the basis of the degree of gait and stance impairment. This assertion can be made because our study provides an objective segmentation of the spectrum of severity of ataxia in SCA3 patients in contrast with previous classifications [11] and reveals their pattern of cerebellar alteration, which is a profile by which these patients show early and severe impairment in gait and stance with mild dysfunction in finger chase, nose-finger tests and sitting stability. Definitions of patterns of cerebellar signs measured by SARA in different ataxias could be helpful for the diagnostic process of patients with progressive ataxia, especially at the start of follow-up. We also suggest that extracerebellar signs of motor system dysfunction, especially the alteration of presynaptic dopamine pathway, and its relationship with impairment of fast hand alternating movements, could add more information to distinguish different ataxias. For instance, a patient with normal DaTSCAN SPECT imaging and very severely altered fast alternating movements is not typical of any SCA3 pattern and other possibilities should be looked for.

Network analysis and heatmap are complementary tools to study cerebellar dysfunction in ataxias. Network analysis studies the relationships of SARA items and clinical parameters in the whole group of patients. In contrast, hierarchical clustering and heatmap study patient by patient and try to order them into different groups following the type and degree of cerebellar dysfunction. The results provided by these techniques may sound contradictory in SCA3 but they are not. Network analysis helps us to understand, that in this sort of ataxia, cerebellar signs are related between them. The heatmap is able to differentiate patterns of cerebellar dysfunctions which classification depends mainly on severity, as it was expected due to the important relationships between signs demonstrated by network analysis. This study provides a method to better understand highly heterogeneous disease profiles in ataxias and other neurological diseases.

## Conclusions

We propose a new approach to address the variability of clinical phenotypes of ataxias from a multivariate point of view. We have tested the usefulness of those tools to improve our understanding about cerebellar dysfunction in SCA3 by means of studying different configurations or patterns in the tests included in SARA and the relationships with other tests of extracerebellar motor alteration. Due to differences in disease duration and other factors, SCA3 patients at start of follow-up in our unit show a high variability in cerebellar phenotype and they can be classified in three different groups of progressive severity based on their impairment in gait and stance. Cerebellar dysfunction is poorly correlated with other motor signs or the length of CAG allelic expansion. Our work demonstrates that DaTSCAN SPECT imaging is frequently abnormal in these patients and weakly correlated with their clinical extrapyramidal signs, suggesting a subclinical affection of the dopaminergic pathway in these patients that affects the clinical expression of cerebellar dysfunction. We suggest applying these approaches to other ataxias and to more numerous groups of SCA3 patients.

## Methods

### Study group

Seventeen (12 men and 5 women) with genetically confirmed diagnosis of SCA3 were included in this study. A standardized clinical interview was carried out at the start of follow up to collect demographic and personal data and to assess the previous clinical course of the disease. In addition genealogical data and previously performed genetic studies were collected.

### Examination

All patients underwent a complete neurological examination that included collection of SARA items scores, taken by one neurologist (either JA or IS). Descriptions of every patient are shown in Table 1. The global SARA score was collected as well as the breakdown of itemized scores (gait, stance, sitting, speech disturbance, right and left finger chase, right and left nose-finger, right and left fast alternating hand movements and right and left heel-shin slide tests) in order to analyse patterns across the group of patients. We collected and analysed left and right scores independently since side asymmetry was expected in cerebellar signs. As clinical indicators of extracerebellar motor system disorder, we used the presence or absence of: pyramidal signs (defined by presence of clonus at triceps surae, Babinski’s and/or Hoffman’s signs), the presence or absence of extrapyramidal signs (defined by presence of hypomimia, rigidity, bradykinesia and/or rest tremor) and the presence of decreased patellar reflexes (DPR). As indicator of abnormalities in presynaptic dopaminergic pathway, we assessed the presence of visually detected abnormalities in DaTSCAN-SPECT imaging after visual evaluation. An expert looked for abnormalities in striatal shape, extent, symmetry and particularly in the intensity of signal. In addition, we also included the intensity of polyneuropathy (severe, moderate, mild, as assessed by nerve conductions studies) as objective indicator of peripheral affection (Additional file 1 Table S1). Motor nerve conduction studies included peroneal, median and ulnar nerves. Sensory nerve conduction was assessed in median, ulnar, sural and superficial peroneal nerves. Electromyography was performed in tibialis anterior and/or extensor digitorum brevis muscle. Upper limb muscles were only occasionally explored.

A set of other clinical variables included age at disease onset (defined by the beginning of symptoms according to the patient’s description), age at the assessment, sex and number of CAG repeats in the expanded allele.

### Descriptive analysis

Median and range were used for quantitative and ordinal variables. Absolute and relative frequencies were calculated for qualitative variables.

### Pattern analysis

Two different approaches were used to classify the clinical profiles of cerebellar dysfunctions of our sample of SCA3 patients. A first approach explored the apparently heterogeneous expression of cerebellar dysfunctions and intended to outline a SCA3 distinguishing pattern referred to alterations in single itemized SARA scores. This approach included: 1) classification of patients by means of a multiple correspondence analysis [27] from which we took the calculated distances (8 first dimensions) to perform a hierarchical clustering analysis, and 2) classification of SARA items scores by hierarchical clustering analysis, using “1- absolute value of Spearman’s rho correlation coefficient” as distance variable. Average was used as grouping criterion in both classifications. To display the results, we used a heatmap (Fig. 1), which is a graphical representation of data where colours are assigned to normalized values (0 will indicate no alteration and 1 will indicate the highest possible alteration in the scale) and parameters and subjects are reordered following a hierarchical clustering classification, shown as dendrograms. This facilitates the reading and detection of the features of the disease “pattern” [28].

As a second approach, a network analysis was conducted in order to understand the inter-correlation of all SARA items scores, non-cerebellar motor signs and a set of other clinical parameters. We used Spearman’s correlation coefficient as weighted edge. The results were represented in a node diagram (Fig. 2) in which only correlations with an absolute value higher than 0.5 were shown. Due to the descriptive nature of this technique, correction for multiple comparisons is not needed [29]. Network analysis has been used in other disciplines, such as econometrics or psychometrics, to describe pairwise relationships between parameters, and might equally well be used to define correlations between SARA items scores, non-cerebellar motor signs and other clinical variables. Network analysis allows processing of highly dimensional information and representation of complex statistical relationships in clear plots without statistical reduction methods.

R language programming was used for data analysis. Specifically, the packages “gplots” and “FactoMineR” were used for the first objective and the package “qgraph” was used to accomplish the second objective.

### Ethical standards

All procedures performed in studies involving human participants were in accordance with the ethical standards of the institutional and/or national research committee and with the 1964 Helsinki declaration and its later amendments or comparable ethical standards. Each participant authorized to participate in the study after an inform consent process.

## Additional file

Additional file 1: Table S1. Definition of severity levels of polyneuropathy. (DOCX 45 kb)

## Abbreviations

DAT: DaTSCAN SPECT imaging
EVO: time of evolution (years)
EXT: extrapyramidal signs
GAI: gait
LAM: left fast alternating hand movements
LFC: left finger chase
LHS: left heel-shin slide
LNF: left nose-finger test
ONS: age at onset (years)
PNP: polyneuropathy
PYR: pyramidal signs
RAM: right fast alternating hand movements
REP: numbers of repeats in expanded allele
RFC: right finger chase
RHS: right heel-shin slide
RNF: right nose-finger test
SIT: sitting
SPE: speech disturbance
STA: stance
WOM: woman
