# Supplementary material for: Patterns of motor signs in spinocerebellar ataxia type 3 at the start of follow-up in a reference unit
Source: Cerebellum Ataxias. 2016 Feb 23;3:4. doi: 10.1186/s40673-016-0042-6 (PMC4763420; doi:10.1186/s40673-016-0042-6)
Supplement: Additional file 1: Table S1. — Definition of severity levels of polyneuropathy. (DOCX 45 kb) [file 40673_2016_42_MOESM1_ESM.docx]

| Level | Defined by the presence of polyneuropathy with most of the explored nerves with an action potential amplitude between |
| --- | --- |
| Mild | 60-90% of LLN |
| Moderate | 60-30% of LLN |
| Severe | <30% of LLN |

Lower limits of normality (LLN) for CMAPs: peroneal (extensor digitorum brevis 2mV), median (abductor pollicis brevis 4 mV) and ulnar (abductor digiti minimi 6mV). Lower limits of SNAPs: median (20μV), ulnar (15μV), sural (6μV) and superficial peroneal nerves(6μV).
